# Characteristics of older patients with postmenopausal osteoporosis who developed loss of muscle mass during the COVID-19 pandemic – a case–control study

**DOI:** 10.1186/s12891-023-06755-6

**Published:** 2023-08-02

**Authors:** Masaru Tanaka, Masahiro Kanayama, Tomoyuki Hashimoto, Fumihiro Oha, Yukitoshi Shimamura, Takeru Tsujimoto, Yuichi Hasegawa, Tsutomu Endo, Hidetoshi Nojiri, Muneaki Ishijima

**Affiliations:** 1 Spine Center, Hakodate Central General Hospital, Hon-Cho 33-2, Hakodate, Hokkaido, 040-8585 Japan; 2 Department of Medicine for Orthopaedics and Motor Organ, Juntendo University Graduate School of Medicine, 1-5-29-4F Yushima, Bunkyo-Ku, Tokyo 113-0034 Japan

**Keywords:** Dual-energy X-ray absorptiometry, Loss of muscle mass, Living alone

## Abstract

**Background:**

Under the restriction of social activities during the coronavirus disease 2019 (COVID-19) pandemic, there was concern about the loss of muscle mass due to a decrease in physical activity for the elderly. The purpose of this study was to investigate the characteristics of older patients with postmenopausal osteoporosis who developed loss of muscle mass during the COVID-19 pandemic in Japan.

**Methods:**

A total of 54 patients with postmenopausal osteoporosis were evaluated in this study. Whole-body dual-energy X-ray absorptiometry was performed pre- and post-COVID-19 pandemic to measure trunk and lower limb muscle mass. At the time of the post-COVID-19 pandemic, we conducted a survey to compare lifestyle before pandemic (the frequency of going out, the frequency of meeting acquaintances or families living apart, regular exercise habits, walking time, family structure), and comorbidities between the muscle mass loss (ML) group and the muscle mass maintenance (MM) group. The ML group consisted of patients with at least a 5% decrease in lower limb muscle mass or trunk muscle mass.

**Results:**

A significant difference was found only for the family structure (*P* = 0.0279); in the ML group, those living alone were the largest group, while in the MM group they were the smallest group.

**Conclusions:**

The ML group was significantly more likely to live alone than the MM group. The current study showed that loss of muscle mass was more common in patients living alone.

## Background

The coronavirus disease 2019 (COVID-19) global pandemic has been a catastrophic event for the entire population, and it has completely changed our lives. Lockdowns have been repeatedly implemented in many countries to prevent the outbreak of COVID-19. In Japan, the government also requested people to stay-at-home, shorten business, telework, etc. (semi-lockdown). Consequently, some older adults were hesitant to go out even when walking or shopping to worry about becoming infected. In addition, opportunities to meet friends and families living apart were thought to have decreased dramatically. These social changes can lead to a decrease in activities in older adults and may result in loss of muscle mass and strength. Low muscle mass and strength cause sarcopenia and frailty [1, 2]. Thus, there has been concern about an increase in the prevalence of sarcopenia or frailty due to loss of skeletal muscle mass under semi-lockdown.

Previous studies have reported that muscle mass peaks in the 30 s and gradually declines with age [3, 4]. Lang et al. reported that in individuals over the age of 50 years, muscle mass is lost at a rate of 1–2% per year and strength at a rate of 1.5–3% per year [5]. Longitudinal studies have shown that in people aged 75 years, muscle mass is lost at a rate of 0.64–0.70% per year in women and 0.80–0.98% per year in men [6]. However, it is not well understood whether muscle mass loss is accelerated in emergencies, such as the COVID-19 pandemic, compared to normal times. We predicted that some older adults would experience muscle mass loss and reduced activities of daily living owing to limited social activity during the COVID-19 pandemic. This study aimed to measure trunk and lower limb muscle mass using whole-body dual-energy X-ray absorptiometry (DXA) in older patients with postmenopausal osteoporosis and to investigate the characteristics of patients who have developed a significant loss of muscle mass during the COVID-19 pandemic in Japan.

## Methods

This was a case–control study. A total of 81 postmenopausal osteoporosis patients underwent whole-body DXA at our institution between December 2019 and March 2020 (pre-COVID-19 pandemic period) to measure trunk and lower limb muscle mass, in addition to regular bone mineral density testing. One year later (post-COVID-19 pandemic period), the patients were administered whole-body DXA and a questionnaire survey. Of the 81 patients, 14 were excluded because they were under 65 years of age at the time of the pre-COVID-19 pandemic. With the exception of 13 patients who developed serious medical disease (multiple myeloma in one, pancreatic cancer in one) and underwent major surgeries that may have affected muscle mass (total knee arthroplasty in one, total hip arthroplasty in two, spine surgery in seven, osteosynthesis for femoral fracture in one) during the follow-up period, the remaining 54 patients with a mean age of 78 (66–94) years were evaluated in this study. No cases of COVID-19 were reported in this study. The institutional review board of our institution approved this study and informed consent was obtained from all participants.

Trunk and lower limb muscle masses were defined as the respective lean mass values measured using DXA. Trunk muscle mass and lower limb muscle mass were compared before and after the COVID-19 pandemic. Participants were divided into groups of patients who had markedly reduced muscle mass and those who did not, and their clinical characteristics were evaluated. Considering previous reports of less than 2% loss of muscle mass per year [5, 6], the muscle mass loss (ML) group consisted of patients with at least a 5% decrease in lower limb muscle mass or trunk muscle mass, and the muscle mass maintenance (MM) group consisted of the other patients.

A questionnaire survey was conducted at the time of the post-COVID-19 pandemic to investigate the lifestyle before pandemic (frequency of going out, frequency of meeting acquaintances or families living apart, regular exercise habits, walking time, family structure) and the Geriatric Depression Scale 5 (GDS-5) [7]. Responses regarding the lifestyle were obtained using recall methods. Patients with two or more points on the GDS-5 were considered to have a depressive tendency [8]. We compared the frequency of going out, meeting acquaintances or families living apart, regular exercise habits, walking time, family structure, and comorbidities including depressive tendency, diabetes mellitus (DM), chronic kidney disease (CKD), and ischemic heart disease (IHD) between the ML and MM groups.

In addition, the changes in the prevalence of sarcopenia before and after the COVID-19 pandemic were examined. To diagnose sarcopenia, hand-grip strength was measured bilaterally, and a higher value was used. Based on the criteria of the Asian Working Group for Sarcopenia, low hand-grip strength < 18 kg and a skeletal muscle mass index (SMI) cut-off value of 5.4 kg/m^2^ were used to define sarcopenia [9]. The ratio between the appendicular skeletal muscle mass measured using DXA and the height squared defines the SMI.

The statistical analyses were performed using JP Pro version 16.0 statistical software (SAS Institute, NC, USA). The Mann–Whitney U test, Wilcoxon signed rank test, Fischer’s exact test, Yates’ chi-squared test, McNemar test, and Pearson’s chi-square test were applied for statistical comparison, and *P* < 0.05 indicated statistical significance.

## Results

In a total of 54 patients, trunk muscle mass, lower limb muscle mass, and hand-grip strength were 16.1 kg, 9.8 kg, and 19.0 kg, respectively, pre-COVID-19 and 16.0 kg, 9.8 kg, and, 18.5 kg, respectively, post-COVID-19. The prevalence of sarcopenia was 20.4% in the pre-COVID-19 pandemic period, and 20.4% in the post-COVID-19 pandemic period (Table 1).

**Table 1:** Changes in muscle mass, hand-grip strength, skeletal muscle mass index, T-score, and prevalence of sarcopenia in all subjects (*n* = 54)

	Pre-COVID-19 pandemic	Post-COVID-19 pandemic	*P*-value
LLMM (kg)	9.8 ± 1.4	9.8 ± 1.2	0.2631^a^
TMM (kg)	16.1 ± 1.7	16.0 ± 1.6	0.8851^a^
ULMM (kg)	3.0 ± 0.4	3.0 ± 0.5	0.5288^a^
Hand-grip strength (kg)	19.0 ± 4.3	18.5 ± 3.7	0.9783^a^
SMI (kg/m^2^)	5.8 ± 0.8	5.8 ± 0.8	0.1539^a^
Lumbar T-score	-1.6 ± 1.3	-1.4 ± 1.3	< 0.0001^a*^
Femoral neck T-score	-2.3 ± 0.8	-2.3 ± 0.8	0.4558^a^
Prevalence of sarcopenia	20.4% (11/54 patients)	20.4% (11/54 patients)	1^b^

*Abbreviations: LLMM* Lower limb muscle mass, *TMM* Trunk muscle mass, *ULMM* Upper Limb muscle mass, *SMI* Skeletal muscle mass index

^a^*P*-value was determined by using the Wilcoxon signed rank test

^b^*P*-value was determined by using the Chi-square test

^*^Statistically significant

Of 54 patients enrolled, 17 patients were in the ML group, and 37 patients were in the MM group. The background characteristics of both groups are presented in Table 2.

**Table 2:** Background characteristics of the muscle mass loss and muscle mass maintenance groups

	ML group	MM group	*P*-value
Age	80.3 ± 6.8	77.1 ± 7.9	0.1622
LLMM (kg)	10.2 ± 1.6	9.6 ± 1.3	0.3328
TMM (kg)	16.1 ± 1.6	16.1 ± 1.8	0.7374
ULMM (kg)	2.9 ± 0.4	3.0 ± 0.5	0.1988
Hand-grip strength (kg)	17.2 ± 3.8	19.7 ± 4.3	0.0290*
BMI (kg/m^2^)	21.5 ± 2.6	23.0 ± 3.8	0.1535
SMI (kg/m^2^)	6.0 ± 0.9	5.7 ± 0.7	0.1621
Lumbar T-score	-1.8 ± 1.2	-1.5 ± 1.3	0.3649
Femoral neck T-score	-2.3 ± 0.9	-2.3 ± 0.8	0.9904

*Abbreviations: ML* Muscle mass loss, *MM* Muscle mass maintenance*, LLMM* Lower limb muscle mass, *TMM* Trunk muscle mass, *ULMM* Upper limb muscle mass, *BMI* Body mass index, *SMI* Skeletal muscle mass index

The *P*-value was determined by using the Mann–Whitney *U* test

^*^Statistically significant

In the ML group, lower limb muscle mass changed from 10.2 kg to 9.6 kg (*P* = 0.0032 in Wilcoxon signed rank test), and trunk muscle mass changed from 16.1 kg to 15.2 kg (*P* < 0.0003 in Wilcoxon signed rank test). The prevalence of sarcopenia in the ML group increased from 17.6% to 29.4% (*P* = 0.1573) (Table 3).

**Table 3:** Changes in muscle mass, hand-grip strength, skeletal muscle mass, T-score, and prevalence of sarcopenia in the muscle mass loss group (*n* = 17)

	Pre-COVID-19 pandemic	Post-COVID-19 pandemic	*P*-value
LLMM (kg)	10.2 ± 1.6	9.6 ± 1.2	0.0032^a^*
TMM (kg)	16.1 ± 1.6	15.2 ± 1.4	< 0.0003^a^*
ULMM (kg)	2.9 ± 0.4	2.8 ± 0.4	0.0887^a^
Hand-grip strength (kg)	17.2 ± 3.8	17.6 ± 3.7	0.4254^a^
SMI (kg/m^2^)	6.0 ± 0.9	5.8 ± 0.8	0.9933^a^
Lumbar T-score	-1.8 ± 1.2	-1.5 ± 1.2	0.0101^a^*
Femoral neck T-score	-2.3 ± 0.9	-2.3 ± 0.8	0.2281^a^
Prevalence of sarcopenia	17.6% (3/17)	29.4% (5/17)	0.1573^b^

*Abbreviations: LLMM* Lower limb muscle mass, *TMM* Trunk muscle mass, *ULMM* Upper limb muscle mass, *SMI* Skeletal muscle mass index

^a^*P*-value was determined by using the Wilcoxon signed rank test

^b^*P*-value was determined by using the McNemar test

^*^Statistically significant

In contrast, in the MM group, the lower limb muscle mass of the MM group changed from 9.6 kg to 9.8 kg (*P* < 0.0001 in Wilcoxon signed rank test), and trunk muscle mass changed from 16.1 kg to 16.3 kg (*P* = 0.2294 in Wilcoxon signed rank test). The prevalence of sarcopenia in the MM group changed from 21.6% to 16.2% (*P* = 0.1573) (Table 4).

**Table 4:** Changes in muscle mass, hand-grip strength, skeletal muscle mass, T-score, and prevalence of sarcopenia in the muscle mass maintenance group (*n* = 37)

	Pre-COVID-19 pandemic	Post-COVID-19 pandemic	*P*-value
LLMM (kg)	9.6 ± 1.3	9.8 ± 1.3	< 0.0001^a^*
TMM (kg)	16.1 ± 1.8	16.3 ± 1.5	0.2294^a^
ULMM (kg)	3.0 ± 0.5	3.1 ± 0.5	0.2294^a^
Hand-grip strength (kg)	19.7 ± 4.3	18.9 ± 3.7	0.9964^a^
SMI (kg/m^2^)	5.7 ± 0.7	5.9 ± 0.7	< 0.0001^a^*
Lumbar T-score	-1.5 ± 1.3	-1.3 ± 1.3	< 0.0001^a^*
Femoral neck T-score	-2.3 ± 0.8	-2.3 ± 0.8	0.7243^a^
Prevalence of sarcopenia	21.6% (8/37)	16.2% (6/37)	0.1573^b^

*Abbreviations: LLMM* Lower limb muscle mass, *TMM* trunk muscle mass, *ULMM* Upper limb muscle mass, *SMI* Skeletal muscle mass index

^a^*P*-value was determined by using the Wilcoxon signed rank test

^b^*P*-value was determined by using the McNemar test

^*^Statistically significant

No significant differences between groups were found for the frequency of going out, the frequency of meeting acquaintances or families living apart, regular exercise habits, walking time, and comorbidities. A significant difference was found only for the family structure (*P* = 0.0279 in Pearson’s chi-square test); in the ML group, those living alone were the largest group, while in the MM group they were the smallest group (Table 5).

**Table 5:** Results of questionnaire survey and comorbidities in the muscle mass loss and muscle mass maintenance groups

	ML group (*n* = 17)	MM group (*n* = 37)	*P*-value
Frequency of going out (times/week)	2.6	2.4	0.8644^a^
Frequency of meeting acquaintances or families living apart (times/month)	2.1	4.2	0.0997^a^
Regular exercise habits	35.2% (6/17)	56.8% (21/37)	0.2411^b^
Walking time (minutes/day)	18 ± 17	32 ± 34	0.1608^a^
Living alone	52.9% (9/17)	21.6% (8/37)	0.0470^b^*
Living together as a couple	11.8% (2/17)	40.5% (15/37)	0.0568^c^
Living together with her child	29.4% (5/17)	37.8% (14/37)	0.7676^b^
Retirement home	5.9% (1/17)	0.0% (0/37)	0.3148^c^
Depressive tendency	47.1% (8/17)	43.2% (16/37)	0.9738^b^
DM	17.6% (3/17)	13.5% (5/37)	0.6961^c^
CKD	47.1% (8/17)	51.4% (19/37)	1^c^
IHD	11.8% (2/17)	13.5% (5/37)	1^c^

*Abbreviations: ML* Muscle mass loss, *MM* Muscle mass maintenance, *DM* Diabetes mellitus, *CKD* Chronic kidney disease, *IHD* Ischemic heart disease

^a^*P*-value was determined by using the Mann–Whitney *U* test

^b^*P*-value was determined by using Yates’ chi-squared test

^c^*P*-value was determined by using Fischer’s exact test

^*^Statistically significant

## Discussion

The semi-lockdown against the COVID-19 pandemic imposed significant restrictions on social activities for the public. In routine medical care, we have recognized that many outpatients, especially older patients, try to stay at home as much as possible because of worry about contracting COVID-19. These experiences led us to suspect loss of skeletal muscle mass in older patients following a decrease in physical activity under semi-lockdown conditions. Our data demonstrated no significant differences between trunk and lower limb muscle masses before and after the COVID-19 pandemic. This result differs from those of previous studies but may be because the subjects were limited to outpatients with postmenopausal osteoporosis. However, we found that some patients developed significant loss of muscle mass. Patients in the ML group had a significantly higher rate of living alone. This result suggests that patients experiencing loneliness and social isolation are more likely to experience a decline in skeletal muscle mass. In previous studies, socially isolated individuals were found to be at an increased risk for the development of cardiovascular disease [10], infectious illness [11], and cognitive deterioration [12]. In addition, objective and subjective social isolation and loneliness are associated with an increased risk of early mortality [13]. Recently, a scoping review demonstrated that most previous studies addressing the effects of loneliness and social isolation revealed associations between social isolation, loneliness, and frailty [14]. These findings indicate that social isolation and loneliness affect various health problems.

Several studies have examined the association between sarcopenia and various diseases. Leenders et al. reported that patients with type 2 diabetes showed a greater decline in muscle mass with age [15]. Avin et al. demonstrated that CKD is associated with a decline in skeletal muscle mass [16]. It has been reported that 20% of older patients with chronic heart failure have sarcopenia [17]. In contrast, the presence of comorbidities including depressive tendency, DM, CKD, and IHD was not associated with loss of muscle mass in our study. In a short period of approximately one year, comorbidities might not be directly related to muscle mass loss if they are stable.

Magnetic resonance imaging (MRI) and computed tomography have been frequently used instead of DXA to measure muscle mass because of their accuracy [18, 19]. However, lower limb lean mass measured by DXA has been found to be highly correlated with MRI measurements of skeletal muscle volume [20]. Recently, trunk lean mass measured by DXA was also found to be correlated with MRI measurements of the paraspinal muscles, which play an important role in supporting the spinal column [21]. These studies suggest that DXA measurements of trunk and lower limb lean mass would be useful for evaluating actual muscle mass.

This study has several limitations. One criticism of this study was its small sample size. To make our data more credible, it is necessary to conduct a large-scale survey. Second, there is an opinion that a cause of muscle mass loss may have occurred as a result of any medical problems. As mentioned earlier, patients who developed serious medical diseases and underwent major surgeries were excluded as much as possible. Therefore, we should have been able to exclude patients with loss of muscle mass due to medical issues. Third, lifestyle status may be inaccurate, as responses were obtained after the pandemic using recall methods.

In conclusion, loss of lower limb muscle mass and/or trunk muscle mass measured by DXA was more common in individuals who lived alone. For older patients, loneliness and social isolation are likely to be closely associated with loss of muscle mass during the COVID-19 pandemic.

## Data Availability

All data supporting our findings are contained within the manuscript.

## Abbreviations

COVID-19: Coronavirus disease 2019
DXA: Dual-energy X-ray absorptiometry
GDS-5: Geriatric Depression Scale 5
DM: Diabetes mellitus
CKD: Chronic kidney disease
IHD: Ischemic heart disease
ML: Muscle mass loss
MM: Muscle mass maintenance
SMI: Skeletal muscle mass index
MRI: Magnetic resonance imaging
